# Adaptation and validation of a gastrointestinal panel to detect diarrheal virus pathogens on a high-throughput qPCR system

**DOI:** 10.1007/s00430-025-00837-z

**Published:** 2025-06-03

**Authors:** Katja Giersch, Dominik Nörz, Moritz Grunwald, Susanne Pfefferle, Lisa Sophie Pflüger, Nicole Fischer, Martin Aepfelbacher, Marc Lütgehetmann

**Affiliations:** Institute of Medical Microbiology, Virology and Hygiene, University Medical Centre Hamburg-Eppendorf (UKE), Martinistraße 52, D-20246 Hamburg, Germany

**Keywords:** Norovirus, Rotavirus, Adenovirus, Sapovirus, Astrovirus, Enterovirus, Infection, Real time polymerase chain reaction, Cobas 5800, Cobas 6800, Cobas 8800, Molecular diagnostics

## Abstract

With around 2 billion cases each year, infectious gastroenteritis remains a worldwide health problem. A major cause of acute gastroenteritis is infection with enteric viruses, which often leads to hospitalization in children and immunocompromised people. We adapted and validated a gastrointestinal qPCR panel, which simultaneously detects the most common enteric viruses: Norovirus GI and GII, Rotavirus, Adenovirus, Sapovirus, Astrovirus and Enterovirus in stool samples on a fully automated, high-throughput system (Roche cobas5800/6800/8800). Limits of detection (LOD), linear range and precision were determined using dilutions of clinical stool samples, which were quantified by digital droplet PCR. Specificity and sensitivity were evaluated using clinical stool samples from patients with diarrhoea and results were compared with commercial CE-IVD qPCR assays. LODs were below 100 for all targets except for Norovirus GI (3,180 copies/ml), Norovirus GII (299 copies/ml) and Rotavirus (851 copies/ml). The assay showed excellent linearity over 5–6 log steps for all pathogens (r^2^: 0.992–0.998). For inclusivity External Quality Assessment samples were correctly identified, and no false positives occurred in exclusivity panels containing 26 bacterial isolates and 12 clinical virus samples. Specificity and sensitivity determined by using 243 patient samples ranged between 98.2 and 100.0% and 85.7–100.0%, respectively. In this study we validated a lab-developed syndromic qPCR assay that reliably detects the seven most common enteric viruses in clinical stool samples. Our assay provides a fast, fully automated and easily scalable high-throughput solution for gastrointestinal routine virus testing and screening in high-risk patient groups and outbreaks.

**Supplementary Information:**

The online version contains supplementary material available at 10.1007/s00430-025-00837-z.

## Introduction

Infectious gastroenteritis remains a worldwide health problem with an estimated 2 billion cases of diarrhea yearly and with being a major cause of emergency department visits and hospitalization [1, 2]. Enteric viruses are a leading cause of gastroenteritis [3] and Norovirus in particular, which is estimated to cause up to 24% cases of acute gastroenteritis, 11–17% of emergency room or hospital visits, and approximately 70 000–200 000 deaths annually [4, 5], and Rotaviruses, which cause more severe gastroenteritis and leading to up to 453 000 deaths annually in children under 5 [6, 7]. Other enteric viruses such as Sapoviruses, Astroviruses and human Adenoviruses account for about 10–20% of community diarrhea cases in children, and can be associated with outbreaks in all age groups [8, 9]. Infection of immunocompromised patients with these viruses can lead to further complications, such as prolonged shedding and long-lasting diarrhea [5].

Conventional methods for identification of a gastrointestinal viral pathogens, such as antigen tests, microscopic examination and culture, are either not applicable for enteric viruses or are time-consuming, costly and often lack sensitivity and specificity [1, 4]. Detection by real time PCR has thus become common practice for these viruses [10, 11]. Currently, diarrheal virus pathogens can be detected by qPCR using several manual laboratory-developed tests and commercial syndromic panels (e.g. Luminex xTAG Gastrointestinal Pathogen Panel [12], Seegene Allplex Gastrointestinal Panel Assays [13]) or low throughput single cartridge based tests (e.g. BioFire FilmArray gastrointestinal test [14], QIAstat‑Dx Gastrointestinal Panel 2 [15]). However, fully automated high-throughput systems offer several advantages such as better reproducibility, a lower risk of contamination and less hands-on time compared to manual PCR workflows [16].

We adapted and validated a laboratory-developed test (LDT) to detect the most common enteric viral pathogens (Norovirus, Rotavirus, Adenovirus, Sapovirus, Astrovirus, Enterovirus) in stool samples by real-time PCR on the Utility Channel (UCT) of the high-throughput cobas 5800/6800/8800 PCR systems.

## Materials and methods

### Design and setup of the diarrheal virus qPCR assay (NRA_SAE_UCT assay)

The diarrheal virus qPCR panel consists of two assays simultaneously detecting Norovirus GI and GII, Rotavirus (A) and Adenovirus (assay 1: NRA_UCT), and Sapovirus, Astrovirus and Enterovirus A-D (assay 2: SAE_UCT). In the following text, we refer to the diarrheal virus qPCR assay as NRA_SAE_UCT assay. To compile the assay, previously published primer/probe sets detecting Norovirus GI [17], Norovirus GII [17, 18], Rotavirus [19], Adenovirus [20, 21], Sapovirus ( [22] and own design), Astrovirus [23] and Enterovirus [24] were selected and modified (table S1). The assays were selected based on compatibility with the UCT Master Mix for cobas5800/6800/8800 systems (Roche, Mannheim, Germany) and a universal PCR run profile, as well as a good clinical performance as described in previous studies [17, 19–26]. Alignments of relevant sequences were collected from Genbank and compared to published primer/probe sequences using Geneious 9.0 (Dotmatics, Boston, USA, data of the alignment 01/2023). Primers and probes were adjusted to increase inclusivity according to recent data bank queries. To prevent formation of primer dimers formations in the multiplexed reaction, primers were modified with 2’O-methyl RNA bases at the 3’-region as indicated in table S1. Probes are either double-quenched, internally quenched, with a C3-spacer at the 3’-end and/or contain locked nuclear acids (LNA; +). For Sapovirus we implemented two different probes to cover Sapovirus genogroup I, II and V. To reduce enhanced background levels and to improve fluorescence levels, these two probes were designed using different fluorescent dyes (Atto 425 and Atto 550, table S1) and were detected on two different channels (1 and 3, table S2). Primers and probes were custom-made by Ella Biotech (Fuerstenfeldbruck, Germany), Biomers (Ulm, Germany) and Integrated DNA Technologies (IDT) (Coralville IA, USA). All oligos used in this study are listed in table S1.

Cobas omni utility channel reagent kits were prepared according to manufacturer’s instructions. Briefly, primer stock (500 µM concentration) and probe stock solutions (100 µM concentration) were added to 10 ml MMX-R2 master mix to obtain final concentrations in the PCR reaction as indicated in table S2. The PCR master mix was filled into utility channel cassettes which were then loaded onto the system to run tests. The cobas5800/6800/8800 system provides a spike-in RNA full-process control, which is added automatically during extraction and is detected in channel 5 (the respective sequence is not disclosed by the manufacturer; see table S2 for the full run protocol).

### Evaluation of analytical performance

Technical performance evaluation for the NRA_SAE_UCT assay was performed according to the new European Union In Vitro Diagnostics Regulation (2017/746 EU IVDR). To evaluate the analytical sensitivity (lower limit of detection, LoD) and linearity of the NRA_SAE_UCT assay clinical samples which were positive for Norovirus GI and GII, Rotavirus, Adenovirus, Sapovirus, Astrovirus and Enterovirus, were used as standards. For Norovirus GI linearity a quantitative synthetic Norovirus GI RNA standard (no. VR-3234S) obtained from ATCC (Manassas, VA, USA) was used.

For quantification of standards from clinical samples, nucleic acids were extracted from selected stool samples on a MagNA-Pure 96 instrument (Roche Diagnostics) and standards were quantified by digital-PCR using the Qiacuity (Qiagen, Hilden, Germany) digital-PCR system according to manufacturer’s instructions. The unit of the standards is digital copies/ml (dcp/ml).

LoD was determined by serial two-fold dilution of standard suspensions using at least eight dilution steps and 21 repeats per dilution. Dilutions were prepared using a Hamilton IVD STARlet liquid handler (Hamilton, Bonaduz, Switzerland).

Linearity was assessed by ten-fold serial dilution according to CLSI guidelines (i.e. linear fit vs. higher-order polynomal fit) of each enteric virus standard (at least six dilution steps, *n* = 5 per dilution) and calculated using Validation Manager software (Finbiosoft, Espoo, Finland).

The within-run and between-day precision was determined by testing on three different days and by using one high positive, one low positive and one negative clinical sample for each enteric virus in triplicates. Within-lab precision was calculated as sum of squares of precision components. Precision was calculated as standard deviation (SD) with coefficient of variation (CV %) according to ANOVA statistics using Validation Manager (Finbiosoft).

For inclusivity 13 EQA samples (INSTAND, Dusseldorf, Germany) from the gastrointestinal virus (#430/ 2023 and 2024) or Norovirus panel (#381/ 2023 and 2024) were analysed using the new NRA_SAE_UCT assay. Moreover, a DNA ultramer (5’- GAT TTG GCC CTC GCC ACC TAC AAT GCC TGG TTC GTA GGT GGT ACA GCT CCA GAC CCA GAG CGC CCC ACT GAA GGT GCA CCC AAA TTA GTG TTT GAG ATG GAG GGC AAT − 3’) was created (Ultramer, manufactured by IDT) and used to verify inclusivity of Sapovirus variants (accession number LC504373 [27]) that are detected by the probe used in channel 1 (Atto 425) but not by the probe used in channel 3 (Atto 550) (see also table S1). For analysis, the ultramer was dissolved in 1 ml of QuantiTect Nucleic Acid Dilution Buffer (Qiagen) and diluted 1:1000 in Roche cobas PCR medium.

An exclusivity study was performed using 26 isolates of different common enteric bacteria (e.g. *E. coli*,* E. faecium*,* E. gallinarum)* and 12 clinical samples containing viruses (e.g. EBV, HSV-1/2, VZV).

### Clinical evaluation

For clinical validation, 243 clinical stool samples from routine testing were directly measured using NRA_SAE_UCT assay on cobas 5800/6800/8800 systems and compared to the Allplex GI-Virus Assay (Seegene, Duesseldorf, Germany), except for Entereovirus where the LightMix Kit (TIB molbiol, Berlin, Germany) was used. Both CE-IVD assays were used according to the manufacturer’s instructions. Discrepant results obtained with the Allplex GI-Virus Assay (Seegene) were resolved using LightMix Kits for Adenovirus, Norovirus GI and GII, Rotavirus, Sapovirus and Astrovirus (TIB molbiol).

Prior to analysis, a swab was directly dipped into the clinical stool sample, transferred to one tube of cobas PCR medium (4.2 ml) (Roche Diagnostics, Rotkreutz, Switzerland) and vortexed (direct swab stool sample preparation method [28]). Samples that returned invalid after analysis were diluted 1:10 with cobas PCR medium (Roche) and re-analysed.

The study was conducted according to the guidelines of the Declaration of Helsinki. This work was conducted in accordance with § 12 of the Hamburg hospital law (§ 12 HmbKHG). The use of anonymized remnant diagnostic samples from patients was approved and informed consent was waived by the ethics committee of the Hamburg Medical Association (PV5626).

Figures were prepared using Validation Manager (Finbiosoft) and GraphPad Prism 10.2.2. (Boston, USA).

## Results

### Alignments of selected assays against current target sequences

Primer/probe sequences were evaluated for mismatches among currently available virus sequences. In total, 6,595 sequences were evaluated for Norovirus GI, 4561 for Norovirus GII, 842 for Rotavirus, 3,124 for Adenovirus (type 1, 7, 19, 41), 402 for Sapovirus (genogroup I, II, V), 520 for Astrovirus and 4,219 for Enterovirus (Enterovirus D68 and A71, echovirus E11 and E18 and poliovirus) (Table 1). For estimation of drop-out risk, sequences were analysed for the fraction of sequences with more than 1 mismatch per oligo, as well as number of mismatches per sequence overall (Table 1).

**Table 1:**
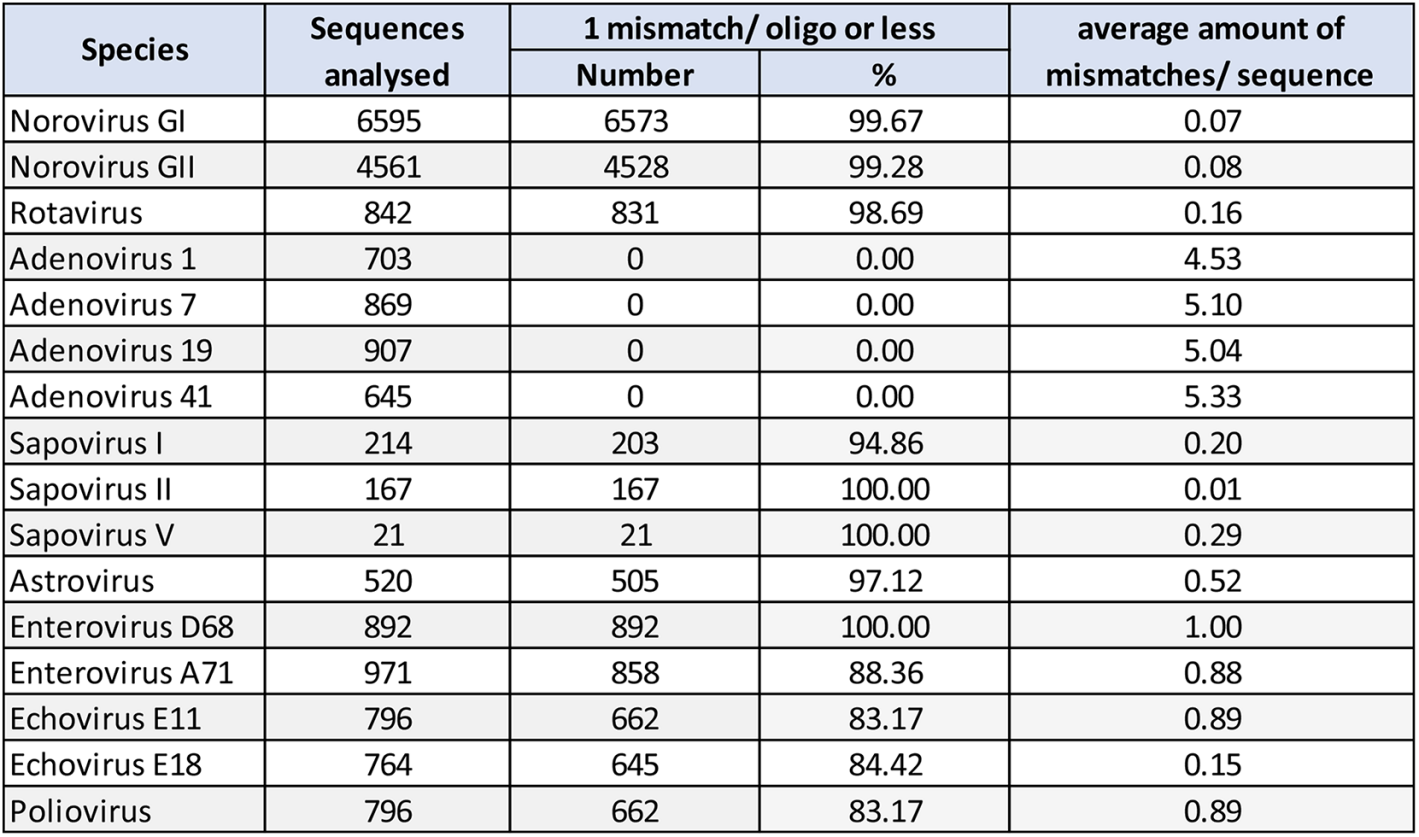
Overview of the in-silico inclusivity review. In case of adenovirus, enterovirus and Sapovirus a representative set of species was aligned to the respective assays. The percentage of sequences with 1 mismatch or less per Oligo, as well as the average number of mismatches per sequence were used as objective criteria for inclusivity

The pan-Adenovirus assay is based on a frequently used design by *Heim et al.* [20]., trying to encompass all human Adenovirus species. It has extensive mismatches against all tested species, but empirically achieves broad inclusivity. We expect variance in ct delays depending on the Adenovirus species present and drop-outs cannot be fully ruled out.

### Analytical sensitivity

**Fig. 1:**
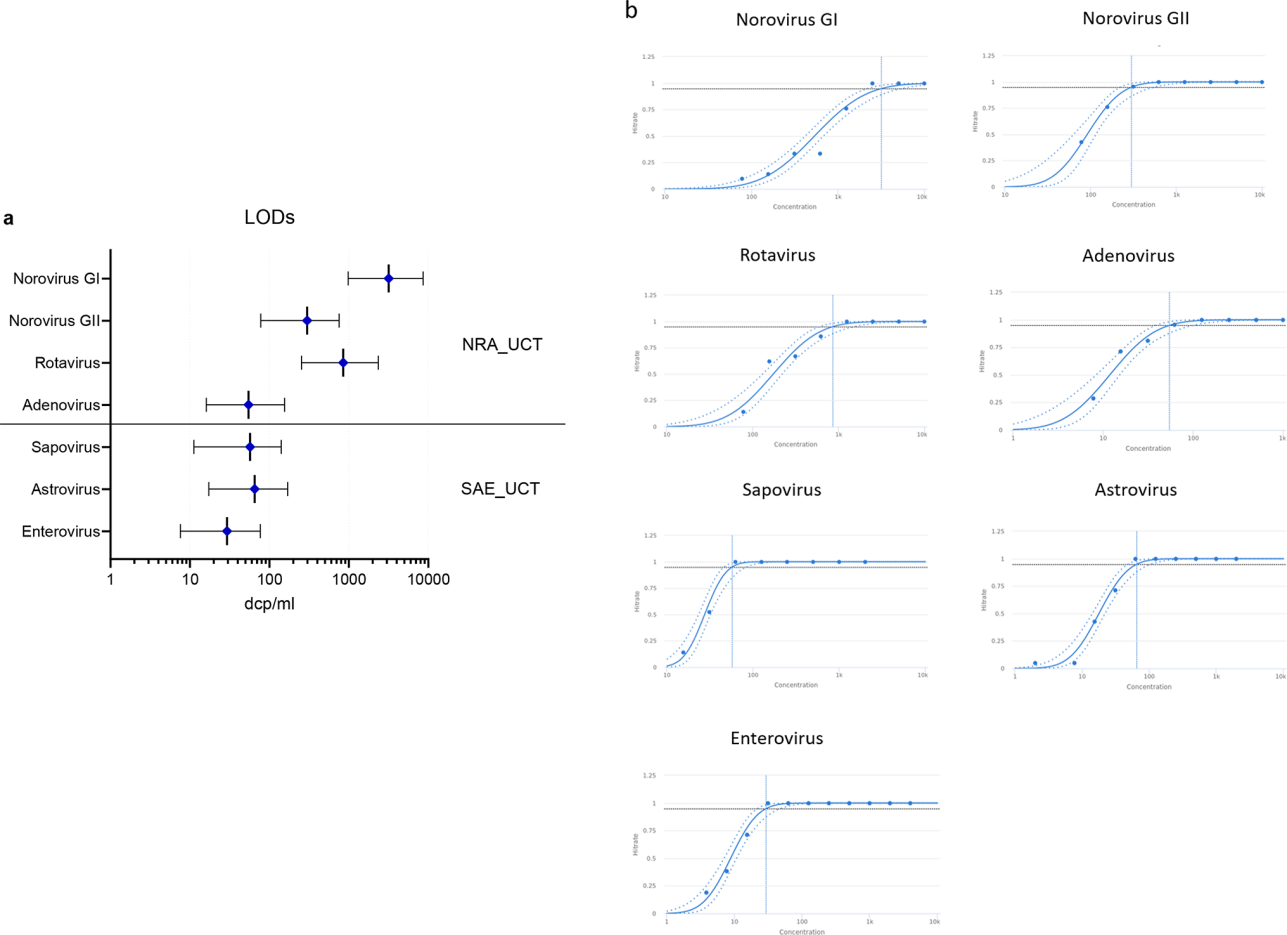
LoDs with 95% confidence intervals in dcp/ml (**a**) and probit curves (**b**) of the analytical sensitivity experiment. Briefly, a 2-fold dilution series of clinical stool samples, which were defined as standards and quantified by digital PCR, was used to determine the 95% probability of detection (21 repeats per dilution step). The concentrations (on dcp/ml) are shown on a logarithmic scale. Confidence intervals are indicated as dash lines. Hit-rates (**b**, y axis) of each concentration are shown table S3

All quantitative standards were determined by digital PCR. Analytic LoDs were evaluated by 95% probit analysis (CLSI EP17-A2) using Validation Manager and demonstrated the following detection limits and confidence intervals (CI): Norovirus GI: 3,180.0 dcp/ml (CI: 2,200.0–5,420.0); Norovirus GII: 299.0 dcp/ml (CI: 221.0–546.0); Rotavirus: 851.0 dcp/ml (CI: 598.0–1500.0); Adenovirus: 54.6 dcp/ml (CI: 38.5–101.0); Sapovirus: 57.0 dcp/ml (CI: 45.8–83.7); Astrovirus: 65.4 dcp/ml (CI: 48.1–105.0); Enterovirus: 29.4 dcp/ml (CI: 21.8–47.6). An overview of LoDs and probit plots are shown in Fig. 1. Concentrations and hit rates are available in table S3.

### Linearity

**Fig. 2:**
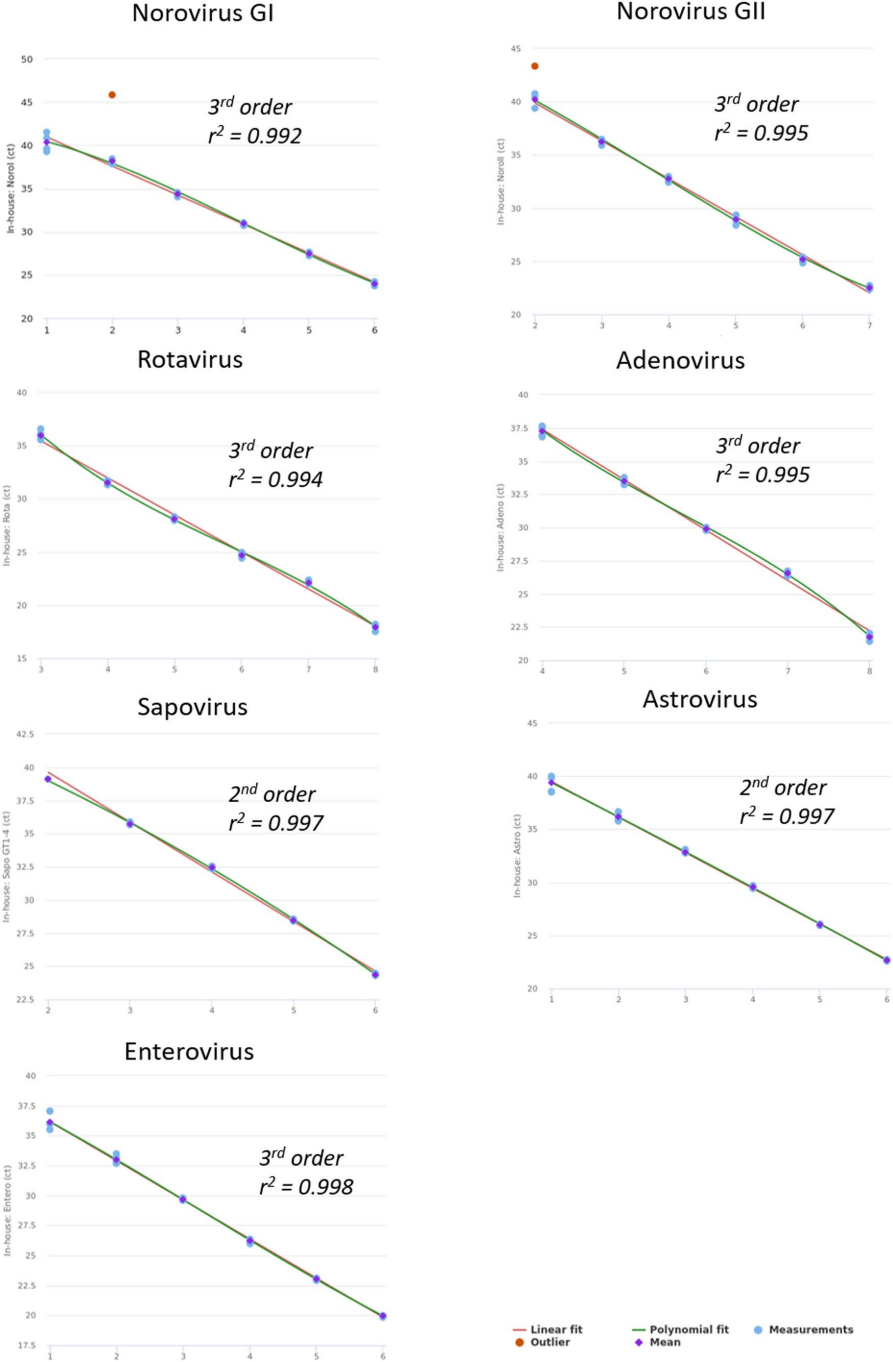
Linearity for each enteric virus. Linearity was determined by serial dilution of standard material (clinical samples). Best fit (polynomial order) and r² is shown in each graph

Linearity was assessed for each pathogen over at least five log-steps through comparing linear fit to higher order polynomial fit mean ct values for each individual dilution step. Linear fit and linear range for each diarrheal virus are shown in Fig. 2; Table 2.

**Table 2:**
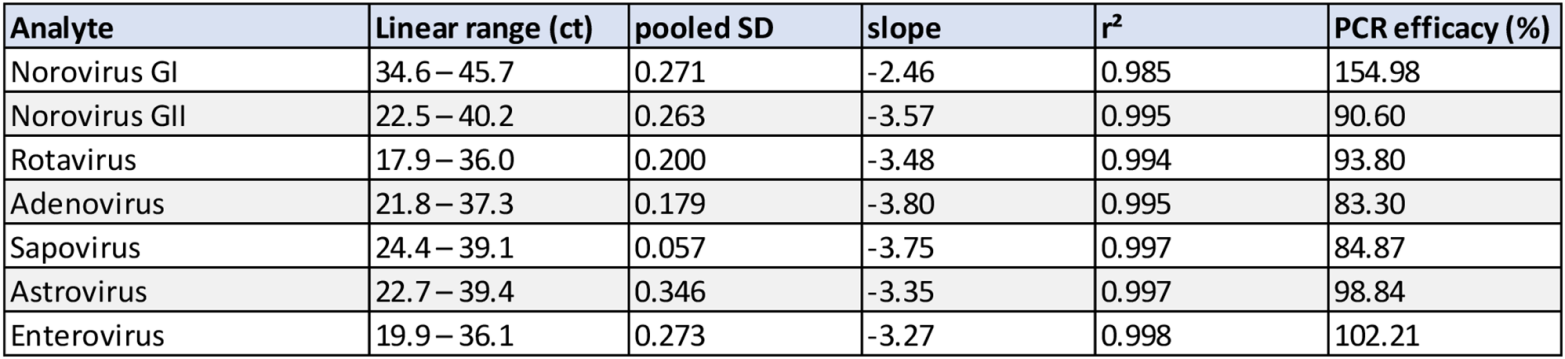
Linearity data for all targets of the NRA_SAE_UCT assay including measurement Ct range, repeatability (pooled SD of absolute difference between linear and higher order polynomial fit), slope, R² and PCR efficacy

### Precision

For analytical precision of each pathogen a high positive, a low positive and a negative clinical sample was measured over three consecutive days. The within-run, between-day and within-lab precision was below 0.83 ct (CV 2.95%), 0.28 (CV 0.79%) and 0.85 ct (CV 3.02%), respectively, for all targets of the NRA_SAE_UCT assay (table S4). Negative clinical samples for each pathogen remained negative on all three days (data not shown).

### Inclusivity and exclusivity

All 13 gastrointestinal virus panel EQA samples (INSTAND) were detected correctly: Norovirus GI: 0/0 positive, 13/13 negative, Norovirus GII: 4/4 positive, 8/8 negative, Rotavirus I: 2/2 positive, 7/7 negative, Adenovirus I: 3/3 positive, 6/6 negative, Sapovirus: 0/0 positive, 8/8 negative, Astrovirus: 2/2 positive, 7/7 negative, Enterovirus: 3/3 positive, 6/6 negative.

The majority of Sapovirus I, II and V variants are detected by the probe (Atto 550) in channel 3 of our assay. However, to also verify the performance and inclusivity of the probe (Atto 425) used in channel 1 we analysed an ultramer DNA of Sapovirus variant LC504373 [27]. As expected, a signal was detected in channel 1 but in channel 3 of the SAE assay using a dilution of the Sapovirus ultramer (ct = 12, data not shown).

No false positives occurred in the exclusivity study containing 26 isolates of different common enteric bacteria and 12 clinical samples positive for 8 different viruses (table S5).

### Clinical performance

The clinical sample set containing 243 stool samples from routine screening was analysed for each target individually. Specificity and sensitivity ranged between 90.8 and 100.0% and 85.7–100.0%, respectively, when using the Allplex™ GI-Virus Assay (Seegene) and LightMix^®^ Kit (TIB molbiol) for enterovirus **(**Table 3a). Of note, the inclusivity for human Adenoviruses of the Allplex™ GI-Virus Assay (Seegene) is limited to species F (Type 40/41) and therefore explaining the high amount of false positive samples with our NRA_SAE_UCT assay (Table 3a).

After resolution of discrepant results using LightMix^®^ Kits (TIB molbiol) for Norovirus GI and GII, Rotavirus, Sapovirus and Astrovirus specificity increased to 98.2–100.0% a (Table 3b). Ct values of remaining false positives ranged between 30.1 and 40.9 (*n* = 10) and of false negatives between 24.5 and 31.4 (*n* = 3) (figure S1).

**Table 3:**
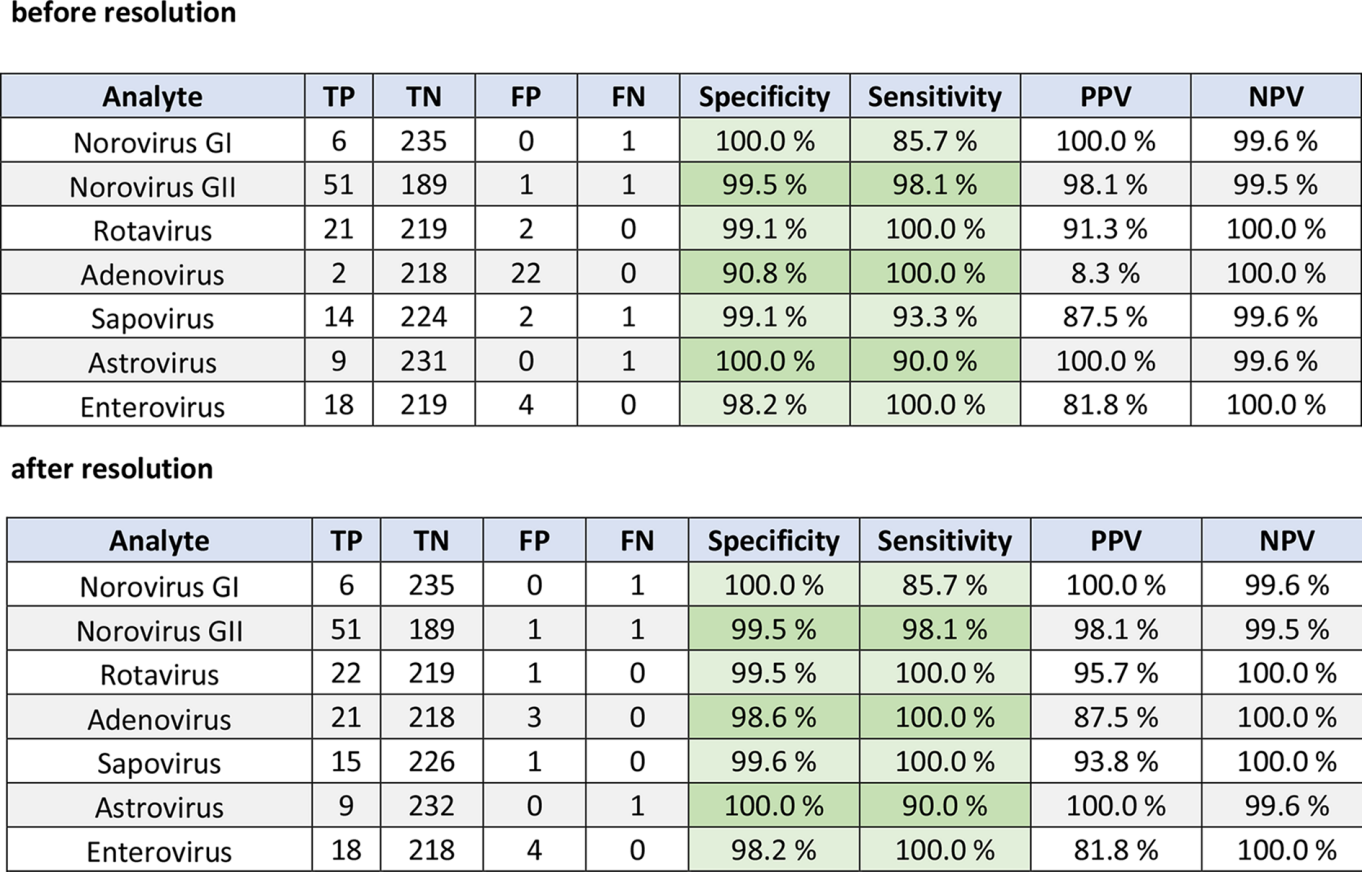
Specificity and sensitivity of each target using clinical stool samples. As reference assay the allplex™ GI-Virus assay (Seegene) detecting Norovirus GI and GII, rotavirus, Sapovirus and astrovirus and LightMix^®^ kit (TIB molbiol) detecting enterovirus were used (a). Discrepant results were resolved using LightMix^®^ kits (TIB molbiol). Discrepant results for enterovirus were not resolved. TP: true positive, TN: true negative, FP: false positive, FN: false negative, PPV: positive predictive value, NPV: negative predictive value

### Review after five months of use in routine diagnostic

**Fig. 3:**
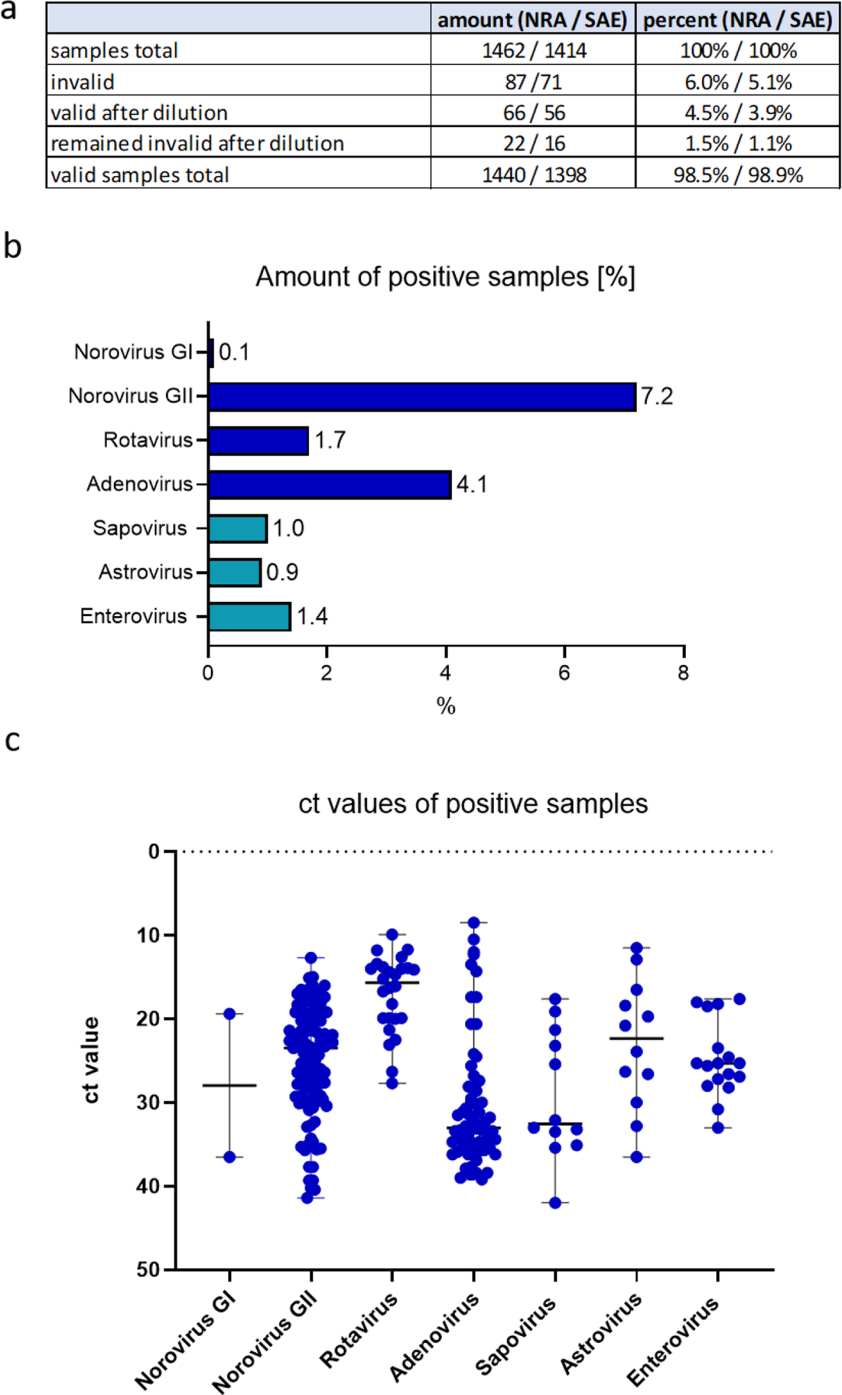
Stool samples analysed using the NRA-SAE_UCT assay in routine diagnostics between February and June 2024. Overview (**a**) and positivity rates (**b**) of all stool samples analysed (in %). Co- and triple- infections are included in the overall amount of positive samples. **c**) ct values of positive samples

The use of the new NRA_SAE_UCT assay in routine diagnostic was evaluated for five months (February - June 2024). During this time 1,462 patient stool samples were analysed for Norovirus, Rotavirus and Adenovirus and 1,414 samples for Sapovirus, Astrovirus and Enterovirus (Fig. 3a). 87 samples (6.0%) and 71 samples (5.1%) of all routine stool samples analysed were invalid, although the direct swab sample method was used to transfer and dilute stool samples in Cobas PCR media (see also Methods) [28]. In real-world conditions, 5.1% (SAE) and 6.0% (NRA) of stool samples are flagged as invalid during PCR analysis due to PCR inhibitors. Invalid samples were diluted (1:10) and re-analysed and the majority (98.7%) showed a valid result (Fig. 3a).

Among the samples analysed 7.8% were Norovirus GII positive and 4.4% were Adenovirus positive, while between 0.9% and 1.8% of stool samples were positive for Rotavirus, Sapovirus, Astrovirus and Enterovirus. Two samples resulted positive Norovirus GI (Fig. 3b). The ct values of all positive samples are shown in Fig. 3c. Interestingly, the majority of ct values for Rotavirus are lower than 20, indicating an acute infection e.g. in children, while most Adenovirus positive samples have ct values higher than 30, which may be an indication of a viral colonization e.g. in immunocompromised patients (Fig. 3c). Ct values for Norovirus GII positive samples are evenly spread from 15 to 40, (Fig. 3c). 18 of the positive samples were either co- or triple-infected with different diarrheal viruses. The most frequent combination (6/18) observed was Rotavirus and Enterovirus (as a co- or triple-infection).

## Discussion

In this study we evaluated a multiplex PCR panel for gastrointestinal viruses on a fully automated high-throughput system (Roche cobas 5800/6800/8800 system). In-house test development on the cobas platform is based on the cobas omni Utility Channel Kit, which includes a spike-in full process control assay. We demonstrate comparable performance to two established, less automated reference CE IVD assay panels.

Diarrhea is a very common symptom with approximately 0.6 episodes per person-year globally and its implications differing widely between high- and low-income environments [29]. Causes for infectious diarrhea include bacterial, viral and parasitic pathogens, however, enteric viruses are of particular concern for healthcare institutions, as they account for a majority of nosocomial outbreaks of infectious gastroenteritis [30]. Norovirus is by far the most common cause of hospital outbreaks and can lead to complications, especially in immunocompromised patients, which may develop prolonged diarrhea, malnutrition, dehydration, allograft dysfunction and can shed the virus for very long periods of time [31]. Therefore, Noroviruses, as well as other commonly found enteric viruses are highly relevant for molecular testing in the clinic.

The qPCR panel presented here combines Norovirus GI/ GII, Rotavirus (A), Adenovirus, Sapovirus, Astrovirus and Enterovirus A-D in two reactions. Our assay showed an excellent linearity over 4–5 log steps for all pathogens (r^2^: 0.985–0.998) and LODs were below 100 for all targets except for Norovirus I (3180 copies/ml), Norovirus II (299 copies/ml) and Rotavirus (851 copies/ml). Noroviruses and other Caliciviruses (i.e. Sapovirus) are notorious for their genetic variability and large number of genotypes, making them difficult targets for diagnostic real-time PCR [32]. To achieve broad inclusivity, we adapted and modified sequences with currently available sequence data from Genbank, to account for genetic diversity as currently possible using publicly available data.

Commercial assay providers regularly limit inclusivity for human Adenoviruses to species F (Type 40/41), as they better correlate with gastrointestinal symptoms [15, 33]. As a result, poor correlation was observed between the NRA_SAE_UCT panel and the Seegene GI Multiplex-panel (90.8%), but not with the Lightmix pan-Adenovirus assay by Tib Molbiol (98.6%). However, broad inclusivity for human Adenoviruses is particularly important in immunosuppressed patients and stem cell transplant recipients, due to the risk of invasive Adenovirus disease, which is detectable in stool prior to full manifestation [34–36].

Similarly, detection of Enteroviruses in stool is often omitted, as Enterovirus infection can manifest in a range of clinical symptoms, not always including gastroenteritis. Enterovirus species and genotypes found in stool samples of symptomatic patients are highly diverse, thus precluding the possibility of a more specific assay [37, 38]. While clinical manifestations of Enterovirus infections are highly diverse, molecular testing of stool samples is always part of the diagnostic workup, thereby inclusion of an Enterovirus target further expands utility of the assay panel [39].

Currently available molecular testing solutions for gastrointestinal pathogens can be broadly divided into rapid point-of-care tests and conventional lab-based tests. Point-of-care tests e.g. include Biofire Filmarray gastrointestinal panel [40] and QIAStat-Dx gastrointestinal panel [15], usually returning results within 1–2 h and also testing for bacterial pathogens. In the conventional field, a growing number of assay panels are available, though usually with a lower degree of automation, e.g. Seegene Allplex gastrointestinal panel, Luminex xTag GPP and BD Max [41]. *Kulis-Horn et al.* have developed an LDT solution for use on the Hologic Panther fusion system, which is a sample-to-result high-throughput platform comparable to the Roche x800 systems. However, this assay only covers Norovirus GI/ GII and Rotavirus [28]. There is an obvious benefit in making enteric virus panels available for different systems, as this will allow for more efficient use of available instruments.

Our new NRA_SAE_UCT assay is being used in routine diagnostics and approximately 1,400 stool samples have been analysed over a five-month period. The most common virus was Norovirus II (7.8%), followed by Adenovirus (4.4%) and Rotavirus (1.8%). 5.6% of all stool samples were initially invalid due to PCR inhibitors, but the majority became valid after 1:10 dilution.

In conclusion, we adapted and validated a broad, laboratory-developed enteric virus qPCR panel for the cobas 5800/6800/8800 system family, enabling fully automated high-throughput processing of clinical stool samples. We demonstrate high analytical and clinical performance comparable to two less automated commercial qPCR panels.

## Electronic supplementary material

Below is the link to the electronic supplementary material.

Supplementary Material 1

## Data Availability

The datasets generated and analysed during the current study are available from the corresponding author on reasonable request.
