# Supplementary material for: Adaptation and validation of a gastrointestinal panel to detect diarrheal virus pathogens on a high-throughput qPCR system
Source: Med Microbiol Immunol. 2025 Jun 3;214(1):28. doi: 10.1007/s00430-025-00837-z (PMC12134015; doi:10.1007/s00430-025-00837-z)
Supplement: Supplementary file 1 — Supplementary Material 1 [file 430_2025_837_MOESM1_ESM.docx]

**Supplementary:**

**Adaptation and validation of a gastrointestinal panel to detect** **diarrheal virus pathogens on a high-throughput qPCR system**

Katja Giersch^1#^, Dominik Nörz ^1#^, Moritz Grunwald^1^, Susanne Pfefferle^1^, Lisa Sophie Pflüger^1^, Nicole Fischer^1^, Martin Aepfelbacher^1^, Marc Lütgehetmann^1*^

1. Institute of Medical Microbiology, Virology and Hygiene, University Medical Centre Hamburg-Eppendorf (UKE), Hamburg, Germany

# shared authorship

* corresponding author

**Correspondence to:**

Dr. Marc Lütgehetmann

Institute of Medical Microbiology, Virology and Hygiene, University Medical Center Hamburg-Eppendorf

Martinistraße 52

D-20246 Hamburg

mluetgeh@uke.de

***Table S1*** *Primer and probe sequences of the NRA_SAE_UTC assay. Sequences are based on previously published assays and modified. Concentrations refer to the final oligo concentrations in each PCR reaction mix. 2’O-methyl-RNA bases are indicated as “OMe-X” and LNA bases as “(+Y)”. ZEN/IBFQ = ZEN–Iowa Black fluorescence quencher, BHQ = Black Hole Quencher*

***Table S2*** *Cobas omni Utility Channel run protocol for the NRA_SAE_UTC assay (2 assays) with internal control (IC). RFI (relative fluorescence increase) thresholds are used for automated result calls*

| NRA_UCT |  |  |  |
| --- | --- | --- | --- |
| **Analyte** | **Concentration** | **Positive results** | **Hitrate** |
| Norovirus GI | 78.1 dcp/ml | 2 / 21 | 0.0952 |
|  | 156 dcp/ml | 3 / 21 | 0.143 |
|  | 313 dcp/ml | 7 / 21 | 0.333 |
|  | 625 dcp/ml | 7 / 21 | 0.333 |
|  | 1,250 dcp/ml | 16 / 21 | 0.762 |
|  | 2,500 dcp/ml | 21 / 21 | 1 |
|  | 5,000 dcp/ml | 21 / 21 | 1 |
|  | 10,000 dcp/ml | 20 / 20 | 1 |
| Norovirus GII | 78.1 dcp/ml | 9 / 21 | 0.429 |
|  | 156 dcp/ml | 16 / 21 | 0.762 |
|  | 313 dcp/ml | 20 / 21 | 0.952 |
|  | 625 dcp/ml | 21 / 21 | 1 |
|  | 1,250 dcp/ml | 21 / 21 | 1 |
|  | 2,500 dcp/ml | 21 / 21 | 1 |
|  | 5,000 dcp/ml | 21 / 21 | 1 |
|  | 10,000 dcp/ml | 20 / 20 | 1 |
| Rotavirus | 78.1 dcp/ml | 3 / 21 | 0.143 |
|  | 156 dcp/ml | 13 / 21 | 0.619 |
|  | 313 dcp/ml | 14 / 21 | 0.667 |
|  | 625 dcp/ml | 18 / 21 | 0.857 |
|  | 1,250 dcp/ml | 21 / 21 | 1 |
|  | 2,500 dcp/ml | 21 / 21 | 1 |
|  | 5,000 dcp/ml | 21 / 21 | 1 |
|  | 10,000 dcp/ml | 20 / 20 | 1 |
| Adenovirus | 7.81 dcp/ml | 6 / 21 | 0.286 |
|  | 15.6 dcp/ml | 15 / 21 | 0.714 |
|  | 31.3 dcp/ml | 17 / 21 | 0.81 |
|  | 62.5 dcp/ml | 20 / 21 | 0.952 |
|  | 125 dcp/ml | 21 / 21 | 1 |
|  | 250 dcp/ml | 21 / 21 | 1 |
|  | 500 dcp/ml | 21 / 21 | 1 |
|  | 1,000 dcp/ml | 20 / 20 | 1 |
|  |  |  |  |
|  |  |  |  |
| SAE_UCT |  |  |  |
| **Analyte** | **Concentration** | **Positive results** | **Hitrate** |
| Sapovirus | 2 dcp/ml | 0 / 21 | 0 |
|  | 3.9 dcp/ml | 0 / 21 | 0 |
|  | 7.8 dcp/ml | 0 / 21 | 0 |
|  | 15.6 dcp/ml | 3 / 21 | 0.143 |
|  | 31.3 dcp/ml | 11 / 21 | 0.524 |
|  | 62.5 dcp/ml | 21 / 21 | 1 |
|  | 125 dcp/ml | 21 / 21 | 1 |
|  | 250 dcp/ml | 21 / 21 | 1 |
|  | 500 dcp/ml | 21 / 21 | 1 |
|  | 1,000 dcp/ml | 21 / 21 | 1 |
|  | 2,000 dcp/ml | 21 / 21 | 1 |
| Astrovirus | 2 dcp/ml | 1 / 21 | 0.0476 |
|  | 3.9 dcp/ml | 0 / 21 | 0 |
|  | 7.8 dcp/ml | 1 / 21 | 0.0476 |
|  | 15.6 dcp/ml | 9 / 21 | 0.429 |
|  | 31.3 dcp/ml | 15 / 21 | 0.714 |
|  | 62.5 dcp/ml | 21 / 21 | 1 |
|  | 125 dcp/ml | 21 / 21 | 1 |
|  | 250 dcp/ml | 21 / 21 | 1 |
|  | 500 dcp/ml | 21 / 21 | 1 |
|  | 1,000 dcp/ml | 21 / 21 | 1 |
|  | 2,000 dcp/ml | 21 / 21 | 1 |
| Enterovirus | 3.9 dcp/ml | 4 / 21 | 0.19 |
|  | 7.8 dcp/ml | 8 / 21 | 0.381 |
|  | 15.6 dcp/ml | 15 / 21 | 0.714 |
|  | 31.3 dcp/ml | 21 / 21 | 1 |
|  | 62.5 dcp/ml | 21 / 21 | 1 |
|  | 125 dcp/ml | 21 / 21 | 1 |
|  | 250 dcp/ml | 21 / 21 | 1 |
|  | 500 dcp/ml | 21 / 21 | 1 |
|  | 1,000 dcp/ml | 21 / 21 | 1 |
|  | 2,000 dcp/ml | 21 / 21 | 1 |
|  | 4,000 dcp/ml | 21 / 21 | 1 |

***Table S3*** *Hit rates of LoD experiment. LoDs were determined by serial dilution of standards, which were generated from diarrheal virus positive clinical samples and quantified by digital PCR. dcp = digital copies*

***Table S4*** *Precision of the all targets of the NRA_SAE_UTC assay. The within-run, between-day and within-lab precision (ANOVA) was determined using a high positive, a low positive and a negative clinical sample in triplicates on three consecutive days. All negative samples were undetected and are not shown in the table. SD: standard deviation (in ct), CV: coefficient of variation (in %)*

***Table S5*** *26 isolates of different common enteric bacteria and 12 clinical samples containing viruses were used for the exclusivity set. No false positives occurred*

| **Virus** | **ct FP** |
| --- | --- |
| Enterovirus | 30.92 |
| Enterovirus | 33.26 |
| Enterovirus | 30.12 |
| Enterovirus | 34.43 |
| Sapovirus | 33.43 |
| Rotavirus | 34.79 |
| Norovirus GII | 39.70 |
| Adenovirus | 40.94 |
| Adenovirus | 35.57 |
| Adenovirus | 37.91 |
|  |  |
| **Virus** | **ct FN** |
| Norovirus GI | 24.8 |
| Norovirus GII | 31.4 |
| Astrovirus | 24.5 |

***Figure S1*** *Ct values of remaining discrepant results in the clinical set. Reference assay was the Allplex™ GI-Virus Assay (Seegene) detecting Norovirus GI and GII, Rotavirus, Sapovirus and Astrovirus and the LightMix® Kit (TIB molbiol) detecting Enterovirus. Discrepant results were resolved using LightMix® Kits (TIB molbiol). FP: false positive, FN: false negative*
